# Induction of Mkp-1 and Nuclear Translocation of Nrf2 by Limonoids from *Khaya grandifoliola* C.DC Protect L-02 Hepatocytes against Acetaminophen-Induced Hepatotoxicity

**DOI:** 10.3389/fphar.2017.00653

**Published:** 2017-09-19

**Authors:** Arnaud F. Kouam, Fei Yuan, Frédéric N. Njayou, Hongtao He, Roméo F. Tsayem, Babayemi O. Oladejo, Fuhang Song, Paul F. Moundipa, George F. Gao

**Affiliations:** ^1^Laboratory of Pharmacology and Toxicology, Department of Biochemistry, Faculty of Science, University of Yaoundé 1 Yaoundé, Cameroon; ^2^CAS Key Laboratory of Pathogenic Microbiology and Immunology, Institute of Microbiology, Chinese Academy of Sciences Beijing, China

**Keywords:** *K. grandifoliola*, limonoids, acetaminophen, hepatoprotection, Mkp-1, Nrf2

## Abstract

Drug-induced liver injury (DILI) is a major clinical problem where natural compounds hold promise for its abrogation. *Khaya grandifoliola* (Meliaceae) is used in Cameroonian traditional medicine for the treatment of liver related diseases and has been studied for its hepatoprotective properties. Till date, reports showing the hepatoprotective molecular mechanism of the plant are lacking. The aim of this study was therefore to identify compounds from the plant bearing hepatoprotective activity and the related molecular mechanism by assessing their effects against acetaminophen (APAP)-induced hepatotoxicity in normal human liver L-02 cells line. The cells were exposed to APAP (10 mM) or co-treated with phytochemical compounds (40 μM) over a period of 36 h and, biochemical and molecular parameters assessed. Three known limonoids namely 17-epi-methyl-6-hydroxylangolensate, 7-deacetoxy-7-oxogedunin and deacetoxy-7R-hydroxygedunin were identified. The results of cells viability and membrane integrity, reactive oxygen species generation and lipid membrane peroxidation assays, cellular glutathione content determination as well as expression of cytochrome P450 2E1 demonstrated the protective action of the limonoids. Immunoblotting analysis revealed that limonoids inhibited APAP-induced c-Jun N-terminal Kinase phosphorylation (p-JNK), mitochondrial translocation of p-JNK and Bcl_2_-associated X Protein, and the release of Apoptosis-inducing Factor into the cytosol. Interestingly, limonoids increased the expression of Mitogen-activated Protein Kinase Phosphatase (Mkp)-1, an endogenous inhibitor of JNK phosphorylation and, induced the nuclear translocation of Nuclear Factor Erythroid 2-related Factor-2 (Nrf2) and decreased the expression of Kelch-like ECH-associated Protein-1. The limonoids also reversed the APAP-induced decreased mRNA levels of Catalase, Superoxide Dismutase-1, Glutathione-*S*-Transferase and Methionine Adenosyltransferase-1A. The obtained results suggest that the isolated limonoids protect L-02 hepatocytes against APAP-induced hepatotoxicity mainly through increase expression of Mkp-1 and nuclear translocation of Nrf2. Thus, these compounds are in part responsible of the hepatoprotective activity of *K. grandifoliola* and further analysis including *in vivo* and toxicological studies are needed to select the most potent compound that may be useful as therapeutic agents against DILI.

## Introduction

Liver is the main site of detoxification and as such, represents the primary target of drug exposure in the body (Kumari and Kakkar, 2012a). DILI is a major clinical problem and public concern worldwide (Jaeschke et al., 2014). It is the case of APAP (*N*-acetyl-para-amino-phenol), a commonly used analgesic and antipyretic drug that is safe at therapeutic dose; but can cause severe hepatotoxicity and even death at an overdose (Nourjah et al., 2006). APAP-hepatotoxicity cannot only bind to its biotransformation, essentially by the isoform 2E1 of cytochrome P450 enzymes to form a reactive metabolite; *N*-acetyl-para benzo-quinone imine (NAPQI), but also to the excessive generation of ROS. Overproductions of NAPQI and ROS cause a depletion of glutathione (GSH) as well as the inactivation of antioxidant enzymes, leading to oxidative stress (Jaeschke et al., 2014). Additionally, NAPQI and ROS if excessive can covalently bind and/or oxidize cellular macromolecules, causing hepatocytes death and liver injury (Xie et al., 2014). Furthermore, several studies have recently demonstrated that oxidant stress activates specific downstream biochemical signaling cascades, which perpetuate APAP-induced hepatotoxicity; including the c-Junc N-terminal kinase (JNK) family (Gunawan et al., 2006; Saito et al., 2010; Xie et al., 2014).

Junc N-terminal kinase proteins are members of the MAPK superfamily that regulate numerous cellular processes and diseases, including liver injury and hepatocytes death (Singh et al., 2009; Saito et al., 2010). Oxidant stress generated during APAP-hepatotoxicity leads to the JNK activation, likely by the upstream kinases, including apoptosis signal-regulating kinases (Nakagawa et al., 2008). Activated JNK amplifies hepatic damages by altering redox status, modulating Bcl-2 protein family activity, and inducing mitochondrial dysfunction (Bajt et al., 2006, 2008, 2011; Saito et al., 2010). Since oxidative stress and JNK activation are emerging as key mediators in APAP-induced liver injury, recent studies have examined up-regulation of antioxidative pathways and endogenous or exogenous JNK inhibitors for their potential therapeutic use (Saito et al., 2010; Wancket et al., 2012; Pang et al., 2016). One such class of endogenous JNK inhibitors is the MAPK phosphatase-1 (Mkp-1) (Keyse, 2000; Wang and Liu, 2007) while the nuclear factor-erythroid 2(NF-E2) related factor-2 (Nrf2) signaling is an example of antioxidative pathways (Niture et al., 2010).

Mkp-1, also referred as DUSP-1 (dual specificity protein phosphatase-1) is the prototype of the Mkp family which preferentially inactivated the stress-induced by MAPK JNK proteins in mammalian cells (Hammer et al., 2006). Kamata et al. (2005) demonstrated that ROS promotes sustained JNK activation and cell death by inhibiting Mkp-1. Moreover, Mkp-1 deficiency enhances APAP hepatotoxicity by prolonging hepatic JNK activation (Wancket et al., 2012). These data provide indications that Mkp-1 maybe a novel endogenous hepatoprotective factor during hepatotoxicity.

Up-regulation of many antioxidant enzymes in the liver is mediated by Nrf2, a transcription factor which plays a pivotal role in the activation of antioxidant gene expression (Niture et al., 2010). Upon cell stimulation, Nrf2 dissociates from its suppressor, Keap-1 and translocates into the nucleus where it binds to the antioxidant response elements (ARE) and promotes the expression of its target genes such as superoxide dismutase (SOD), CAT and glutathione *S*-transferase (GST) (Jaiswal, 2004). It has been shown that Nrf2 protects liver against several xenobiotic through transcriptional up-regulation of antioxidant enzymes (Chen et al., 2013; Pang et al., 2016), suggesting that activation of Keap1-Nrf2 pathway may also be a novel strategy to prevent DILI.

Several herbal medicines and their active constituents have been shown to protect liver against the aforementioned pathological processes (Madrigal-Santillán et al., 2014). It is the case of *Khaya grandifoliola*, a plant belonging to the family of Meliaceae, widely distributed from western Africa to Guinean coast. In Cameroon, *K. grandifoliola* is used in traditional medicine for the treatment of jaundice and others related liver diseases (Moundipa et al., 2002). Ethnobotanical surveys conducted in Nigeria also reported that the plant is used in folk medicine for the treatment of malaria, anemia, arthritis, convulsion and fever (Odugbemi et al., 2007; Olowokudejo et al., 2008). Pharmacological studies on this plant have demonstrated anti-malarial (Makinde et al., 2006), antibacterial (Stephen et al., 2009), anti-anemic (Adeyemi and Gbilade, 2006), anti-inflammatory (Falodun et al., 2009) and antifungal (Onifade, 2006) activities. Phytochemical studies have led to the isolation and characterization of 11 limonoids including grandifotane A (Yuan et al., 2010), methylangolensate and gedunin which showed anti-plasmodial effects (Bickii et al., 2000; Makinde et al., 2006). In addition, its hepatoprotective properties have been demonstrated and, a fraction with highly promising activity has been isolated (Njayou et al., 2013, 2015, 2016). However, there are no available reports showing the hepatoprotective molecular mechanism of its active ingredients. Taking into account the complex pathophysiology of APAP-induced hepatotoxicity, this study was designed to investigate the protective mechanism of active compounds isolated from *K. grandifoliola* in APAP-induced oxidative damage in L-02 cell, a hepatocyte cell line, by assessing their effect on the expression of some proteins involved in APAP-cell death mechanism and cellular antioxidant defense system.

## Materials and Methods

### Chemical Compounds and Reagents

Acetaminophen was purchased from MedChem Express (Monmouth Junction, NJ 08852, United States); Thiazolyl Blue Tetrazolium Bromide, α-Keto-glutaric Acid, L-Alanine, Thiobarbituric Acid, Trichloroacetic Acid, 2′-7′-Dichlorodihydrofluorescein diacetate (H_2_DCFDA), silymarin, JNK inhibitor SP600125, MITOISO2 – Mitochondria Isolation Kit were purchased from Sigma–Aldrich (St. Louis, MO, United States); M-PER Mammalian Protein Extraction Reagent, NE-PER^®^ Nuclear and Cytoplasmic Proteins Extraction Kit, Halt protease inhibitor cocktail EDTA-Free 100X, Pierce bicinchoninic acid (BCA) Proteins Assay Kit, SuperSignal West Pico Chemiluminescent Substrate were all purchased from Thermo Fisher Scientific (Rockford, IL, United States). Rabbit polyclonal anti-CYP2E1 antibody (1:1500 dilution) was purchased from Sino Biological Inc. (Beijing, China); Rabbit polychonal anti-phospho-JNK1/JNK2 and JNK2 antibodies (1:1000 dilution) were purchased from Signalway Antibody (Baltimore, MD, United States); Rabbit polyclonal anti-MKP-1, Nrf2, Keap-1, AIF, COX IV, Lamin B and mouse polyclonal anti-Bax antibodies (all 1:1000 dilution) were purchased from Beijing Biosynthesis Biotechnology CO., LTD. (Beijing, China); Mouse monoclonal anti-βactin primary antibody (1:5000 dilution), horseradish peroxidase-conjugated goat anti-rabbit and anti-mouse IgG AP-linked secondary antibodies (1:2000 dilution) were purchased from Santa Cruz Biotechnology (Santa Cruz, CA, United States); TRIzol^®^ Reagent was purchased from Ambion Life Technologies (Carlsbad, CA, United States); First-Strand cDNA Synthesis Kit was purchased from Promega (Madison, WI, United States); iTaq Universal SYBR Green Supermix Kit was purchased from Bio-Rad Laboratories (Hercules, CA, United States); All primers of the genes of interest were synthetized by TSINGKE Biological Technology Company (Beijing, China). All others reagent used in this study were of analytical grade.

### Preparation of Plant Crude Extracts, Fractions and Sub-Fractions

Stem barks of *K. grandifoliola* were collected in June 2015 in Foumban (West Cameroon). The botanical identification of the plant was done at the Cameroon National Herbarium, where voucher specimen is kept under the reference number 23434 YA. *K. grandifoliola* most active fraction, namely KgF25 (methylene chloride/methanol 75:25, v/v) was prepared as previously described (Njayou et al., 2015, 2016). Therefore, 8 g of KgF25 were subjected to silica gel 60 (particle size 40–63 μm) column chromatography (column: d × h = 3.5 cm × 65 cm) and gradient of hexane/acetyl acetate (100:0 v/v to 0:100 v/v), then acetyl acetate/methanol (10:90 v/v to 0:100 v/v) were used for elution. The 47 chromatographic fractions containing crystalline solid obtained were analyzed by thin layer chromatography (TLC) and pooled into 5 sub-fractions namely KgF25sf1 (41 mg); KgF25sf2 (67 mg); KgF25sf3 (19 mg); KgF25sf4 (105 mg); and KgF25sf5 (55 mg) based on the similarity of their TLC profiles.

### High Performance Liquid Chromatography (HPLC) Analysis and Purification

Following the preliminary results, the most active sub-fractions were analyzed by HPLC-ACN (Acetonitrile)-Standard-Method and compounds were purified by HPLC-ACN-Standard-Purify-Method. We used a Series 1200 Liquid Chromatograph (Agilent Technologies, Folsom, CA, United States) equipped with a vacuum degasser, a quaternary pump, an autosampler and a Diode-Array-Detector (DAD) connected to Agilent ChemStation software. An Eclipse XDB-C8 column (9.4 mm × 250 mm, 5 μm particle size) was used. The mobile phase was (A) water; (B) acetonitrile. The flow rate was 1 mL/min. The elution conditions were: B, 0–15 min, increasing gradient from 0 to 30% B; 15–20 min, linear gradient 100% B; 20–25 min, linear gradient 30% B. The system operated at 28°C and the injection volume of each sample dilute in methanol (1 mg/mL) was 5 μL. The detection wavelength was kept at 254 nm.

### Spectral Analysis of Purified Compounds

High-Resolution Mass Spectra (HRMS) of each pure sample dissolved in methanol was measured on an Agilent 1200 HPLC/6520 High-resolution mass spectrometer. NMR of each pure sample dissolved in DMSO (5 mg/mL) was recorded on BRUKER AV 500 spectrometer.

### Cell and Culture Conditions

The normal human liver cell line L-02, (Cell Bank, Type Culture Collection of Institute of Microbiology, Chinese Academy of Sciences, Beijing, China) derived from an adult human normal liver were used. Cells were cultured in 100 mm dish and maintained in high glucose Dulbecco’s Modified Eagle’s Medium supplemented with 10% fetal bovine serum, L-glutamine (2 mM), penicillin (100 IU/mL), streptomycin (100 μg/mL) and amphotericin B (0.25 μg/mL) in an atmosphere of 5% CO_2_ at 37°C.

### Cells Treatment: General Procedure

In this study, silymarin and JNK-inhibitor SP600125 were used as reference compounds. APAP, silymarin, sub-fractions of KgF25 and isolated compounds were diluted in 20% DMSO [20% DMSO in phosphate-buffer saline (PBS)]. JNK-inhibitor SP600125 was dissolved in 100% DMSO. L-02 cells (approximately 2.10^5^ cells/ml) in triplicate were seeded into 24-well plate labeled as control, APAP, standard and test (references or plant sample + APAP) and incubated for 24 h. Thereafter, the medium was replaced with fresh medium and cells were incubated in absence (control group) or in presence of APAP (APAP group), or in presence of APAP and references or plant sample (test groups) for different time points (6; 12; 24; and 36 h) depending on the downstream analysis.

### Determination of the Toxic Concentration of APAP to Be Used

Cells were incubated in presence of APAP at the final concentrations of 0; 5; 10; 15; 20; and 30 mM. After 36 h, cell viability was measured using 3-(4, 5-dimethylthiosol-2-yl)-2, 5-diphenyl-2H-tetrazolium bromide kit (MTT; Sigma–Aldrich) according to the manufacturer’s instructions and the cell membrane integrity was assessed by measuring ALT activity released into the incubation medium using a calibration curve according to Reitman and Frankel (1957). Cell viability was expressed as percentage of the control and the half lethal concentration (LC_50_) of APAP was determined and used as toxic concentration for the following experiments.

### Hepatoprotective Activity Screening of Plant Sub-Fractions and Dose-Response Study of the Selected Active Sub-Fractions and Isolated Compounds

Cells were treated simultaneously with APAP (LC_50_) and plant sub-fractions or silymarin at the final concentration of 100 μg/mL for the hepatoprotective activity screening. After 36 h, cell viability and cell membrane integrity were assessed as abovementioned. Selected active sub-fractions and silymarin were tested at the final concentration of 25; 50; and 100 μg/mL for the dose-response study. Isolated compounds were then tested at 0; 10; 20; 30; and 40 μM and JNK inhibitor SP600125 at the final concentration of 0; 5; 10; 15; and 20 μM in order to minimize the effect of DMSO in APAP metabolism. After end time point, cell viability and membrane integrity were assessed and the half efficient concentrations (EC_50_) determined.

### Propidium Iodide (PI) Staining

Cells were simultaneously treated with APAP and isolated compounds or JNK inhibitor at the determined concentration. After 36 h, cells were washed with cold PBS and stained with 10 μg/mL of PI solution for 5 min. Cells were then stained with 1 μg/mL of 4′,6-diamino-2-phenylindole (DAPI) solution for 25 min and image were recorded with a fluorescence microscope (Advanced Microscopy Group).

### Measurement of Intracellular ROS Level and Lipid Membrane Peroxidation

Intracellular ROS were measured according to reported method (Ji et al., 2009). Briefly, cells were treated with 20 μM H_2_DCFDA, APAP (10 mM) and isolated compounds or JNK inhibitor at the determined concentrations for 36 h. After treatment, supernatant was collected and cells were washed with PBS, immediately lysed in an appropriate lysis buffer, the whole cell lysates were centrifuged (10,000 *g*, 5 min, 4°C) and aliquot of lysate was used for fluorescence measurement at excitation 485 ± 20 nm, emission 525 ± 20 nm in a black wall with clear bottom 96-well plate using a spectrophotometer (SpectraMax M5, Molecular Devices) and presented as percentage of control. Lipid membrane peroxidation was evaluated through the malondialdehyde (MDA) level in the cellular supernatant by the thiobarbituric acid (TBA) method by using its molar extinction coefficient (ε_MDA_ = 1.56 × 10^5^ M^-1^.Cm^-1^) as described by Buege and Aust (1978).

### Measurement of Cellular GSH Content

After treating cells with APAP and isolated compounds or JNK inhibitor for the indicated time, cells were harvested and lysed in lysis buffer. The whole cell lysates were centrifuged (10,000 *g*, 5 min, 4°C). The supernatant was collected and the GSH concentration was determined by using its molar extinction coefficient (ε_GSH_ = 13,600 M^-1^.Cm^-1^) by the 5,5-dithio-bis (2-nitrobenzoic acid) (DTNB) assay as described by Ellman (1959).

### Protein Extraction and Subcellular Fractionation

Cells were simultaneously treated with APAP and plant sub-fractions, silymarin, isolated compounds or JNK inhibitor at the determined concentration and incubated for different time points. Afterward, total proteins were extracted using M-PER Mammalian Protein Extraction Reagent (Thermo Scientific) containing 0.2% Halt protease inhibitor cocktail EDTA-Free 100X (Thermo Scientific); cytosolic and nuclear protein were extracted using NE-PER^®^ Nuclear and Cytoplasmic Proteins Extraction Kit (Thermo Scientific); mitochondrial and cytosolic fractions were prepared with MITOISO2 - Mitochondria Isolation Kit (Sigma–Aldrich); protein concentration in each sample was quantified with Pierce BCA Proteins Assay Kit (Thermo Scientific). All procedures were performed according to each manufacturer’s instructions.

### Western Blot Analysis

Equal amount of protein in Laemmli loading buffer (10 μl containing ≈ 50 μg of protein) was separated by 12 % Sodium-Dodecyl-Sulfate Poly-Acrylamide Gel Electrophoresis (SDS-PAGE) and electro-transferred into a Nitrocellulose Blotting Membrane (GE Healthcare, Life Science, Germany). The membrane were blocked with 5% w/v dehydrated skimmed milk in Tris-Buffered Saline Tween-20 (TBST: 10 mM Tris-HCl; 150 mM NaCl; 0.05% tween-20; pH 7.6); incubated overnight at 4°C with primary antibodies, rinsed, and then incubated 1 h at 25°C with horseradish peroxidase-conjugated secondary antibodies. Membrane was stained SuperSignal West Pico Chemiluminescent Substrate (Thermo Fisher Scientific, United States) and detection was performed by Enhanced Chemiluminescent Method which combines MicroChemi Unit and GelCapture Software. Densitometry analysis of the protein bands was performed using ImageJ Software.

### RNA Isolation and cDNA Synthesis

L-02 cells were simultaneously treated with APAP and isolated compounds or JNK inhibitor and incubated for the indicated time. After treatment, Total RNA was extracted from cells using TRIzol^®^ Reagent (Ambion, Life Technologies) according to the manufacturer’s instruction. RNA concentration and purity were determined by reading the absorbance at 230; 260; and 280 nm by using a ND-2000 NanoDrop Spectrophotometer (Thermo Scientific). The 260/280 ratio of our RNA preparation ranged from 1.8 to 2.1 while the 260/230 ratio were greater than 2. These values indicate good RNA quality and therefore could be used for reverse transcription. First-strand synthesis cDNA was synthetized in the thermo cycler (Eastwin, Life Science) using Moloney Murine Leukemia Virus Reverse Transcriptase (M-MLV RT, Promega, Woods Hollow Road Madison, United States) as directed by the manufacturer.

### Quantitative Real-Time Polymerase Chain Reaction (qRT-PCR) Analysis

Real-time qPCR was performed in Applied Biosystems 7500 System using iTaq^TM^ Universal SYBR^®^ Green Supermix (Bio-RAD Laboratories, Hercules, CA, United States) according to the manufacturer’s instruction. Relative expression of target genes was normalized to the endogenous gene (GAPDH) used as internal control, analyzed by the 2^-ΔΔ*C*_T_^ method using GenEX Software and given as ratio compared to the control group. All primers of interest genes were synthetized by TSINGKE Biological Technology Company (Beijing, China). Their sequences are presented in the **Table 1**.

**Table 1:** Primer sequences used for quantitative real-time polymerase chain reaction (qRT-PCR).

Genes	Primer Sequence (5′____3′)	
	Forward	Reverse
CAT	AGGCCAGTCCTGACAAAATG	GAATCTCCGCACTTCTCCAG
SOD1	GAAGGTGTGGGGAAGCATTA	ACATTGCCCAAGTCTCCAAC
GST	TTGGCCTCCTGTATTCCTTG	AGCCAACTGGATGCTGAGTT
MAT1A	TAGGGACTGACCAGCAGCTT	AGGGACCAGGGAAAGAGAAA
GAPDH	CGACCACTTTGTCAAGCTCA	AGGGGTCTACATGGCAACTG


*CAT, catalase; SOD1, superoxide dismutase1; GST, glutathione-*S*-Transferase; MAT1A, methionine adenosyltransferase 1A; GAPDH, glyceraldehyde-3-phosphate deshydrogenase.*

### Statistical Analysis

Results are presented as mean ± standard deviation (SD) of three independent experiments in triplicate. Comparisons between the mean values of various treatments groups were analyzed by one-way analysis of variance (ANOVA) followed by the Bonferroni’s *post hoc* test whenever significant differences were observed between the variances. Comparisons were made between untreated group (DMSO control group) and intoxicated group (APAP-intoxicated group), and between APAP-intoxicated group and treated groups (APAP + tested or reference compounds). Differences between compared groups were considered significant for *p* < 0.05. Analyses were performed using Prism 5.03 statistical software (Graph Pad Inc.).

## Results

### HPLC Fingerprint of Selected Active Sub-Fractions and Spectral Analysis of Purified Compounds

**FIGURE 1:**
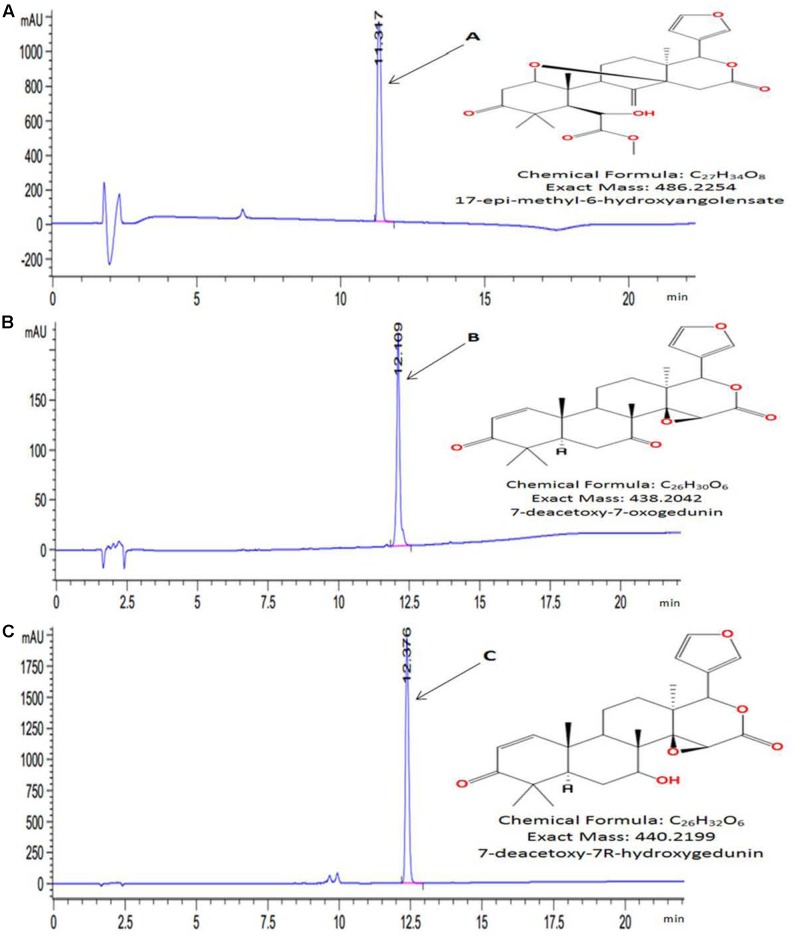
High performance liquid chromatography (HPLC) chromatogram and chemical structures of purified compounds. **(A)** 17-epi-methyl-6-hydroxyangolensate; **(B)** 7-deacetoxy-7-oxogedunin and **(C)** 7-deacetoxy-7R-hydroxygedunin. Compounds were purified by HPLC-ACN-Standard-Purify-Method. Exact mass and chemical structures were determined by HRMS and ^1^H and ^13^C NMR, respectively.

High Performance Liquid Chromatography chromatogram of KgF25sf1 (Supplementary Figure S1A) showed several peaks with different retention time. The higher peak (retention time: 11.22 min) was designated as compound A (C-A). HPLC chromatogram of KgF25sf2 (Supplementary Figure S1B) showed two main peaks with the retention time at 12.05 and 12.33 min and were designated as compound B (C-B) and (compound C) (C-C) respectively. C-A, C-B and C-C were purified and characterized. Comparison of theirs HRMS and NMR spectral data (Supplementary Figures S2–S10) with data literature (Atta-ur-Rahman et al., 2008; Tanaka et al., 2011, 2012) allowed for the unambiguously identification of three known limonoids (**Figure 1**) as 17-epi-methyl-6-hydroxyangolensate, 7-deacetoxy-7-oxogedunin and 7-deacetoxy-7R-hydroxygedunin, respectively, for C-A, C-B and C-C.

### APAP-Induced Cell Death and Loss of Membrane Integrity in L-02 Hepatocytes

**FIGURE 2:**
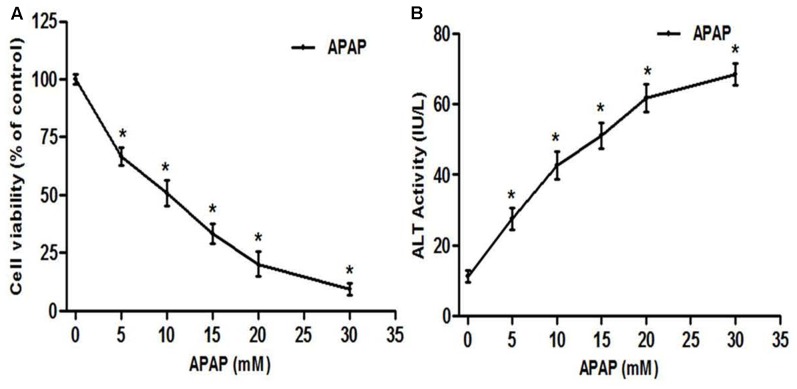
Acetaminophen (APAP) induced cell death and loss of membrane integrity in L-02 hepatocytes. Cells were treated with various concentration of APAP (5–30 mM) for 36 h. **(A)** Cell viability indicating dose-response of APAP toxicity; **(B)** loss of membrane integrity indicated by ALT activity found in the culture medium. Values are means ± SD of three independent experiments in triplicate. ANOVA analysis: *F*(5,30) = 377.7, *P* < 0.0001 **(A)** and *F*(5,30) = 251.7, *P* < 0.0001 **(B)**. ^∗^Values significantly different compared to control (0 mM) (*P* < 0.05) using Bonferroni’s test.

As a normal hepatic cell line, L-02 cells are commonly used in hepatotoxicity studies induced by various xenobiotic (Xiao et al., 2014; Pang et al., 2016). Extra cellular level of ALT activity, a cytosolic enzyme is routinely used to evaluate the membrane integrity during APAP toxicity study *in vitro* (Xie et al., 2014). We evaluated the toxicity of APAP in L-02 cells by performing a dose-dependent study with APAP concentration range from 5 to 30 mM. Exposure of L-02 hepatocytes to APAP for 36 h dose-dependently resulted in significant (*p* < 0.05) loss of cell viability (**Figure 2A**) and increase cellular ALT leakage into the incubation medium (**Figure 2B**). The LC_50_ was found to be 9.80 ± 2.61 mM, and therefore, 10 mM of APAP was chosen as toxic concentration for the following experiment in this work.

### Hepatoprotective Activity Screening of KgF25 Sub-Fractions

**FIGURE 3:**
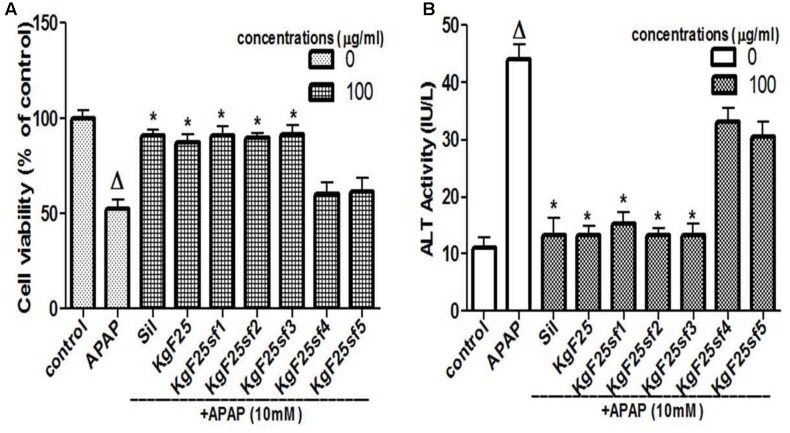
Hepatoprotective screening activity of plants sub-fractions. L-02 cells were treated without or with APAP, or co-treated with APAP and KgF25 sub-fractions or silymarin for 36 h. After treatment, cell viability **(A)** and ALT activity **(B)** leakage into the incubation were determined. Values are means ± SD of three independent experiments in triplicate. ANOVA analysis: *F*(8,45) = 76.47, *P* < 0.0001 **(A)** and *F*(8,45) = 180.3, *P* < 0.0001 **(B)**. ^Δ^Values significantly different compared to control group (*P* < 0.05); ^∗^values significantly different compared to APAP intoxicated group (*P* < 0.05) using Bonferroni’s test. Sil: silymarin; KgF25: methylene chloride/methanol (75:25, v/v) fraction of *K. grandifoliola*; KgF25sf1: sub-fraction 1 of KgF25; KgF25sf2: sub-fraction 2 of KgF25; KgF25sf3: sub-fraction 3 of KgF25; KgF25sf4: sub-fraction 4; of KgF25, KgF25sf5: sub-fraction 5 of KgF25.

The sub-fractions were tested against APAP-induced hepatotoxicity. As presented in **Figures 3A,B**, respectively, sub-fractions KgF25sf1, KgF25sf2, and KgF25sf3 significantly (*p* < 0.05) maintained cell viability and inhibited the release of ALT from the cell. These activities were not statistically different from that of silymarin, a well-known hepatoprotective flavonoids mixture. The above sub-fractions, being the most active, have been selected for the next part of the study.

### Dose-Dependent Protective Effect of Active Sub-Fractions and Isolated Limonoids against APAP-Induced Toxicity

**FIGURE 4:**
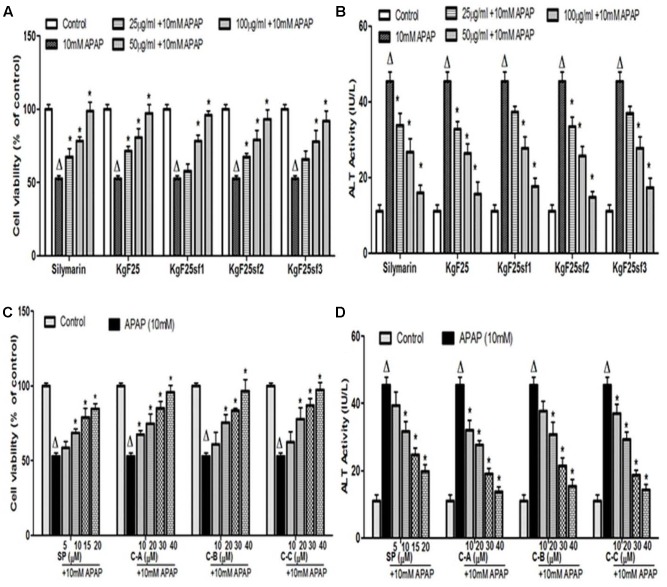
Dose-dependent protective effect of active sub-fractions and isolated limonoids. Cells were treated without or with APAP (10 mM), or co-treated with APAP (10 mM) and active sub-fractions or silymarin (25; 50; and 100 μg/mL), isolated limonoids (10; 20; 30; and 40 μM) or JNK inhibitor (5; 10; 15; and 20 μM) for 36 h. **(A,B)** Effect of active sub-fractions on cell viability and ALT leakage, respectively. **(C,D)** Effect of isolated limonoids on cell viability and ALT leakage, respectively. Values are means ± SD of three independent experiments in triplicate. ANOVA analysis: *F*(6,14) = 33.82, *P* < 0.0001 **(A)**; *F*(6,14) = 77.74, *P* < 0.0001 **(B)**; *F*(5,12) = 48.96, *P* < 0.0001 **(C)**; and *F*(5,12) = 128.3, *P* < 0.0001 **(D)**. ^Δ^Values significantly different compared to control group (*P* < 0.05); ^∗^values significantly different compared to APAP intoxicated group (*P* < 0.05) using Bonferroni’s test. KgF25: methylene chloride/methanol (75:25, v/v) fraction of *K. grandifoliola*; KgF25sf1: sub-fraction 1 of KgF25; KgF25sf2: sub-fraction 2 of KgF25; KgF25sf3: sub-fraction 3 of KgF25; C-A: 17-epi-methyl-6-hydroxyangolensate; C-B: 7-deacetoxy-7-oxogedunin; C-C: 7-deacetoxy-7R-hydroxygedunin; SP: JNK inhibitor SP600125.

**FIGURE 5:**
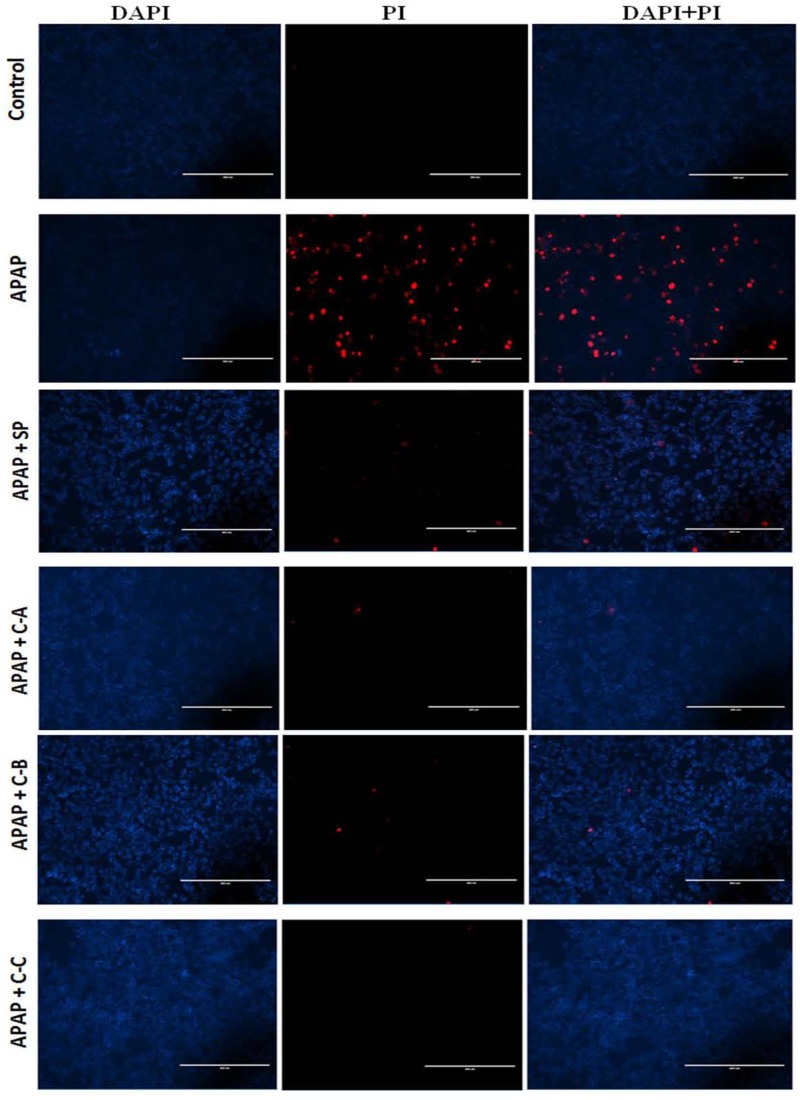
Propidium Iodide staining showing protective effect of isolated compounds against APAP-induced cell death. Cells were treated without or with APAP (10 mM), or co-treated with APAP (10 mM) and isolated compounds (40 μM) or JNK inhibitor (20 μM) for 36 h. After treatment, death cells were stained with PI (red) and DAPI (blue) was used to label the total number of cells. Each image recorded with fluorescence microscope represents one of three independent experiments in triplicate. SP: JNK inhibitor SP600125; C-A: 17-epi-methyl-6-hydroxyangolensate; C-B: 7-deacetoxy-7-oxogedunin; C-C: 7-deacetoxy-7R-hydroxygedunin.

Incubation of cells with APAP (10 mM) for 36 h resulted in a significant (*p* < 0.05) decrease of cell viability (**Figure 4A**) and increase of ALT (**Figure 4B**) activity leakage into the incubation medium. When hepatocytes were co-treated with active sub-fractions or silymarin at various concentrations (25; 50; and 100 μg/mL), a concentration-dependent protective and inhibitory effects on cell viability (**Figure 4A**) and ALT leakage (**Figure 4B**) was observed. Likewise, a similar effect on these parameters (**Figures 4C,D**) was observed when cells were co-treated with isolated limonoids (10; 20; 30; and 40 μM) or JNK inhibitor SP600125 (5; 10; 15; and 20 μM). The EC_50_ were 19.04 ± 3.42 μM, 20.89 ± 3.68 μM, 17.40 ± 2.79 μM, and 13.76 ± 2.37 μM, respectively, for compound A (17-epi-methyl-6-hydroxyangolensate), compound B (7-deacetoxy-7-oxogedunin), compound C (7-deacetoxy-7R-hydroxygedunin) and JNK inhibitor SP600125. Although the EC_50_ of JNK inhibitor was lower than those of isolated limonoids, JNK inhibitor offered only partial protection (cell viability = 85.66 ± 3.51%) while limonoids from *K. grandifoliola* showed almost total protection when added at 40 μM (cell viability ≥ 95%) as confirmed by PI cell death staining (**Figure 5**). When active sub-fractions or isolated compounds were added at 100 μg/mL or 40 μM, the viability of hepatocytes were not statistically different from those of control group and the levels of extracellular ALT activity were lower. Based on this observation, these concentrations were chosen for the following experiments.

### Isolated Compounds Attenuated and Rescued APAP-Induced GSH Depletion, via Inhibition of Cytochrome P450 2E1 (CYP2E1) Expression

**FIGURE 6:**
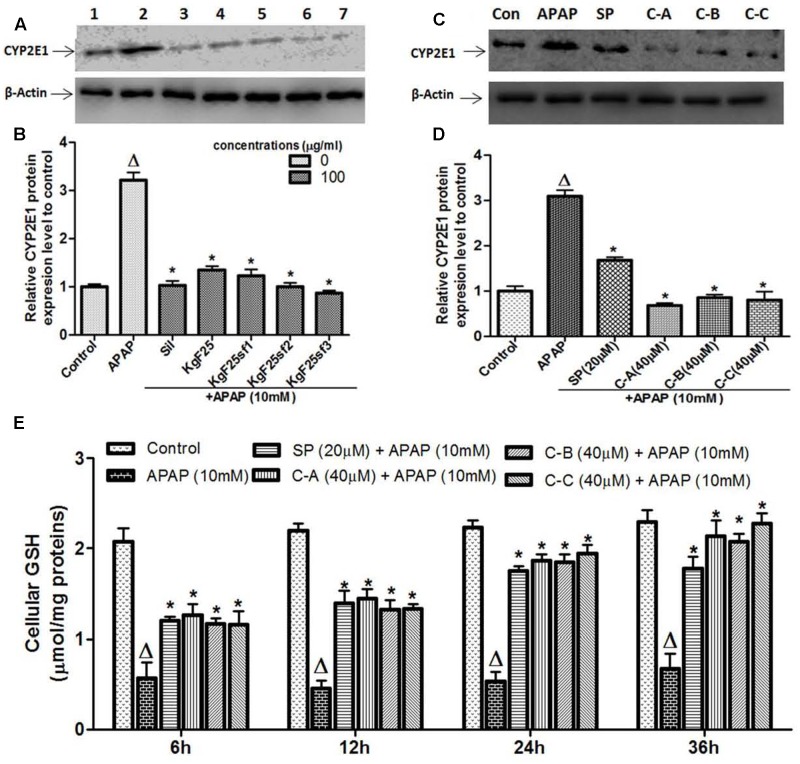
Active sub-fractions and isolated limonoids down-regulated CYP2E1 proteins expression and rescued APAP-induced GSH depletion. Cells were treated without or with APAP (10 mM), or co-treated with APAP (10 mM) and active sub-fractions or silymarin (100 μg/mL), isolated compounds (40 μM) or JNK inhibitor (20 μM) for 6 h. After treatment, total proteins were extracted from cells and CYP2E1 expression was determined by western blotting. β-actin was used as loading control. Each blot represents one of three independent experiments. **(A,C)** Effect of active sub-fractions and isolated compounds on CYP2E1 expression, respectively. **(B,D)** Densitometry analysis of blots, respectively, for active sub-fractions and isolated compounds. **(E)** Effect of isolated compounds on cellular GSH. L-02 cells were treated without or with APAP (10 mM), or co-treated with APAP (10 mM) and isolated compounds (40 μM) or JNK inhibitor (20 μM) for 6, 12, 24, and 36 h. After each time point, total proteins were extracted and GSH contents were quantified. Values are means ± SD of three independent experiments in triplicate. ANOVA analysis: *F*(6,14) = 200.9, *P* < 0.0001 **(B)**; *F*(5,12) = 219.6, *P* < 0.0001 **(D)**; and *F*(5,12) = 59.88, *P* < 0.0001 **(D)**. ^Δ^Values significantly different compared to control group (*P* < 0.05); ^∗^values significantly different compared to APAP intoxicated group (*P* < 0.05) using Bonferroni’s test. Lane1: control; Lane2: APAP; Lane3: silymarin+APAP; Lane4: KgF25+APAP; Lane5: KgF25sf1+APAP; Lane6: KgF25sf2+APAP; Lane7: Kgf25sf3+APAP. Sil: silymarin; KgF25: methylene chloride/methanol (75:25, v/v) fraction of *K. grandifoliola*; KgF25sf1: sub-fraction 1 of KgF25; KgF25sf2: sub-fraction 2 of KgF25; KgF25sf3: sub-fraction 3 of KgF25; SP: JNK inhibitor SP600125; C-A: 17-epi-methyl-6-hydroxyangolensate; C-B: 7-deacetoxy-7-oxogedunin; C-C: 7-deacetoxy-7R-hydroxygedunin.

Acetaminophen hepatotoxicity is initiated by its biotransformation by CYP2E1 to the electrophile NAPQI, and overexpression of CYP2E1 plays a critical role in this process (Jaeschke et al., 2002). To evaluate whether the protective effect of active sub-fractions and isolated compounds was related to the expression of CYP2E1, the protein expression of CYP2E1 was determined by western blot analysis after co-treatment of cells with APAP (10 mM) and active sub-fractions (100 μg/mL) or isolated compounds (40 μM). The constitutive up-regulation of CYP2E1 expression by APAP treatment was significantly (*p* < 0.05) down-regulated when cells were co-treated with active sub-fractions (**Figures 6A,B**) or isolated compounds (**Figures 6C,D**). This effect was comparable to that observed in silymarin (100 μg/mL) treated cells (**Figure 6A**). NAPQI, the reactive metabolite of APAP can react with the sulfhydryl group of cysteine in GSH, leading to its depletion and therefore enhance cellular oxidative stress (Jaeschke et al., 2012). As shown in **Figure 6E**, exposure to 10 mM APAP significantly (*p* < 0.05) reduced cellular GSH levels within 6 h, followed by further depletion during the next 30 h. Co-treatment of L-02 cells with isolated limonoids significantly (*p* < 0.05) attenuated GSH depletion after 6 h, followed by progressive restoration during the next 30 h. After 36 h, cellular GSH levels in co-treated cells were comparable to that of normal untreated cells.

### Isolated Limonoids Attenuated APAP-Induced ROS Generation and Lipid Peroxidation

**FIGURE 7:**
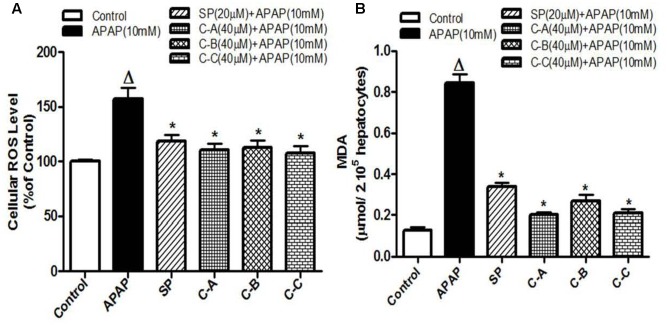
Limonoids from *K. grandifoliola* attenuated APAP-induced oxidative stress in L-02 cells. Cells were treated without or with APAP (10 mM), or co-treated with APAP (10 mM) and isolated compounds (40 μM) or JNK inhibitor (20 μM) for 36 h. After treatment, cellular ROS level **(A)** and MDA concentrations **(B)** in the incubation medium were measured. Values are means ± SD of three independent experiments in triplicate. ANOVA analysis: *F*(5,12) = 28.25, *P* < 0.0001 **(A)** and *F*(5,12) = 303.0, *P* < 0.0001 **(B)**. ^Δ^Values significantly different compared to control group (*P* < 0.05); ^∗^values significantly different compared to APAP intoxicated group (*P* < 0.05) using Bonferroni’s test. SP: JNK inhibitor SP600125; C-A: 17-epi-methyl-6-hydroxyangolensate; C-B: 7-deacetoxy-7-oxogedunin; C-C: 7-deacetoxy-7R-hydroxygedunin.

Increase production of intracellular ROS occurring after GSH depletion plays an important role in APAP-induced injury (Jaeschke et al., 2002). **Figure 7A** depicts the effect of APAP-induced intracellular ROS generation in L-02 cells and its significant annihilation by co-treatment with isolated limonoids from *K. grandifoliola*. Hepatocytes stressed with APAP showed significant (*p* < 0.05) increase in ROS generation as compared to untreated cells. Co-treatment with isolated compounds (40 μM) or JNK inhibitor (20 μM) significantly (*p* < 0.05) decreased ROS level as compared to APAP-treated cells. Overproduction of ROS initiates the process of lipid peroxidation in cell membranes and causes the destruction of cellular integrity as well as cell death (Kumari and Kakkar, 2012b). Lipid peroxidation was measured by estimating the concentration of MDA, an end product of lipid peroxidation into the incubation medium. As presented in **Figure 7B**, oxidative stress induced by free radical due to APAP in the hepatocytes caused a significant (*p* < 0.05) increase of MDA production. However, the MDA formation was significantly attenuated when cells were co-treated with isolated limonoids (40 μM) or JNK inhibitor (20 μM).

### Active Sub-Fractions and Isolated Compounds Prevented APAP-Induced JNK Activation and Mitochondrial Translocation of p-JNK in L-02 Hepatocytes

**FIGURE 8:**
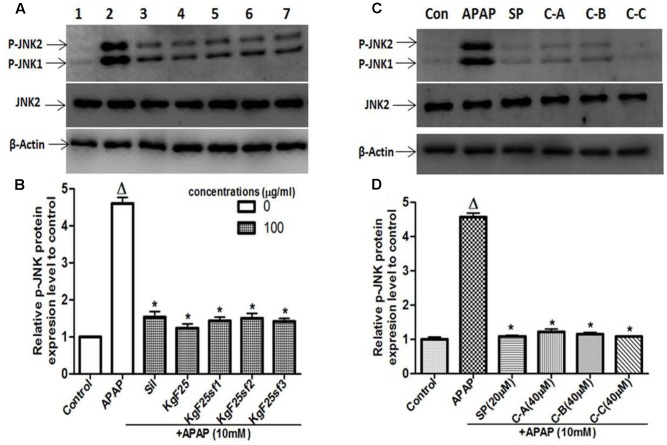
Active sub-fractions and isolated limonoids prevented APAP-induced JNK activation. Cells were treated without or with APAP (10 mM), or co-treated with APAP (10 mM) and active sub-fractions or silymarin (100 μg/mL), isolated limonoids (40 μM) or JNK inhibitor (20 μM) for 6 h. After treatment, total proteins were extracted from cells and the lysates were probed for JNK2 and p-JNK by western blotting. β-actin was used as loading control. Each blot represents one of three independent experiments. **(A,C)** Effect of active sub-fractions and isolated compounds on JNK activation, respectively. **(B,D)** Densitometry analysis of blots, respectively, for active sub-fractions and isolated compounds. Values are means ± SD of three independent experiments in triplicate. ANOVA analysis: *F*(6,14) = 322.0, *P* < 0.0001 **(B)** and *F*(5,12) = 1162.0, *P* < 0.0001 **(D)**. ^Δ^Values significantly different compared to control group (*P* < 0.05); ^∗^values significantly different compared to APAP intoxicated group (*P* < 0.05) using Bonferroni’s test. Lane1: control; Lane2: APAP; Lane3: silymarin+APAP; Lane4: KgF25+APAP; Lane5: KgF25sf1+APAP; Lane6: KgF25sf2+APAP; Lane7: Kgf25sf3+APAP. Sil: silymarin; KgF25: methylene chloride/methanol (75:25, v/v) fraction of *K. grandifoliola*; KgF25sf1: sub-fraction 1 of KgF25; KgF25sf2: sub-fraction 2 of KgF25; KgF25sf3: sub-fraction 3 of KgF25; SP: JNK inhibitor SP600125; C-A: 17-epi-methyl-6-hydroxyangolensate; C-B: 7-deacetoxy-7-oxogedunin; C-C: 7-deacetoxy-7R-hydroxygedunin.

Recently, JNK activation and the mitochondrial translocation of p-JNK have been well established to play a major role in APAP-induced hepatotoxicity (Gunawan et al., 2006; Hanawa et al., 2008; Xie et al., 2014). To explore the effects of isolated compounds on JNK mediating cell death, total proteins were initially extracted 6 h after treating L-02 cells with 10 mM APAP or co-treated with APAP and active sub-fractions (100 μg/mL) or limonoids (40 μM). The lysates were probed for JNK2 and p-JNK by western blotting. Administration of APAP alone resulted in massive p-JNK while co-treatment with active sub-fractions (**Figure 8A**) or isolated compounds (**Figure 8B**) significantly (*p* < 0.05) inhibited JNK activation.

**FIGURE 9:**
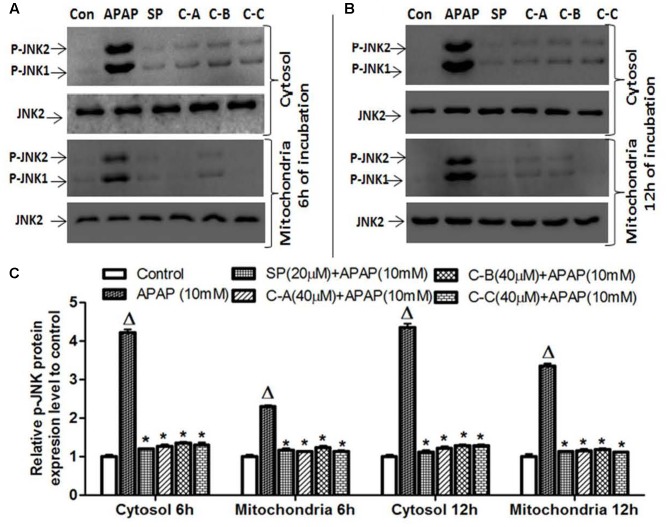
Limonoids from *K. grandifoliola* prevented mitochondrial p-JNK translocation. Cells were treated without or with APAP (10 mM), or co-treated with APAP (10 mM) and isolated limonoids (40 μM) or JNK inhibitor (20 μM) for 6 and 12 h. After treatment, p-JNK level was detected into the cytosolic and mitochondrial fractions by western blotting. JNK2 was used as internal control. Each blot represents one of three independent experiments. **(A,B)** Effect of isolated compounds on JNK activation, respectively, at 6 and 12 h after treatment. **(C)** densitometry analysis of blots. Values are means ± SD of three independent experiments in triplicate. ANOVA analysis: *F*(5,12) = 1119.0, *P* < 0.0001 and *F*(5,12) = 1841.0, *P* < 0.0001 **(C)**. ^Δ^Values significantly different compared to control group (*P* < 0.05); ^∗^values significantly different compared to APAP intoxicated group (*P* < 0.05) using Bonferroni’s test. SP: JNK inhibitor SP600125; C-A: 17-epi -methyl-6-hydroxyangolensate; C-B: 7-deacetoxy-7-oxogedunin; C-C: 7-deacetoxy-7R-hydroxygedunin.

Activated JNK translocate into mitochondria, leading to mitochondrial dysfunction and oxidant stress (Jaeschke et al., 2012). To determine whether isolated limonoids affect p-JNK translocation into mitochondria, cytosolic and mitochondrial fractions were prepared from L-02 cells and JNK activation was evaluated at 6 and 12 h after treatment. Similar to what was observed in total cell lysates, 10 mM APAP exposure caused JNK activation in the cytosol at 6 and 12 h (**Figures 9A,B**). We observed in the mitochondria of APAP treated cells a modest, but significant (*p* < 0.05) increase in p-JNK 6 h after APAP exposure (**Figure 9A**) and massive p-JNK at 12 h (**Figure 9B**). Co-treatment with isolated compounds prevented JNK activation in the cytosol at 6 and 12 h and its translocation into mitochondria (**Figures 9A,B**). These effects were comparable to those observed in JNK inhibitor treated cells.

### Limonoids from *K. grandifoliola* Prevented Mitochondrial Bax Translocation and Release of AIF from Mitochondria to the Cytosol

**FIGURE 10:**
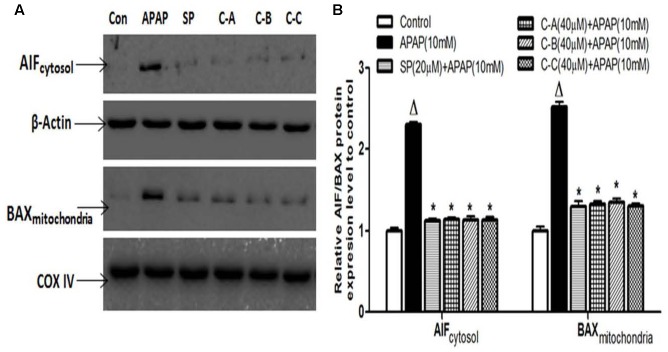
Isolated compounds attenuated mitochondrial Bax translocation and release of AIF from mitochondria to cytosol. Cells were treated without or with APAP (10 mM), or co-treated with APAP (10 mM) and limonoids (40 μM) or JNK inhibitor (20 μM) for 12 h. After treatment, AIF and Bax protein levels **(A)** were detected into the cytosolic and mitochondrial fractions by western blotting. β-actin and COX IV (cytochrome oxidase IV) were used as loading control, respectively, for the cytosolic and mitochondrial fraction, respectively. Each blot represents one of three independent experiments. **(B)** Densitometry analysis of blots. Values are means ± SD of three independent experiments in triplicate. ANOVA analysis: *F*(5,12) = 671.2, *P* < 0.0001 and *F*(5,12) = 317.5, *P* < 0.0001 **(B)**. ^Δ^Values significantly different compared to control group (*P* < 0.05); ^∗^values significantly different compared to APAP intoxicated group (*P* < 0.05) using Bonferroni’s test. SP: JNK inhibitor SP600125; C-A: 17-epi-methyl-6-hydroxyangolensate; C-B: 7-deacetoxy-7-oxogedunin; C-C: 7-deacetoxy-7R-hydroxygedunin.

Amplification of the mitochondrial oxidant stress by the translocation of p-JNK triggers the opening of the MPT pore in the mitochondria, followed by the mitochondrial Bax (Bcl_2_-associated X protein) translocation. These effects are responsible for the translocation of mitochondrial intermembrane proteins such as AIF and endonuclease G to the nucleus as main cause of nuclear DNA damage and cell death after APAP overdose (Bajt et al., 2008, 2011; Saito et al., 2010). To investigate the effect of isolated limonoids in these events, the release of AIF into the cytosol and mitochondrial translocation of Bax were observed by immunoblotting in APAP-intoxicated cells 12 h after exposure (**Figures 10A,B**). Co-treatment of cells with isolated compounds (40 μM) or JNK inhibitor (20 μM) significantly (*p* < 0.05) prevented the mitochondrial translocation of Bax and the release of AIF into the cytosol (**Figures 10A,B**).

### Isolated Limonoid Up-Regulated Mkp-1 Protein Expression

**FIGURE 11:**
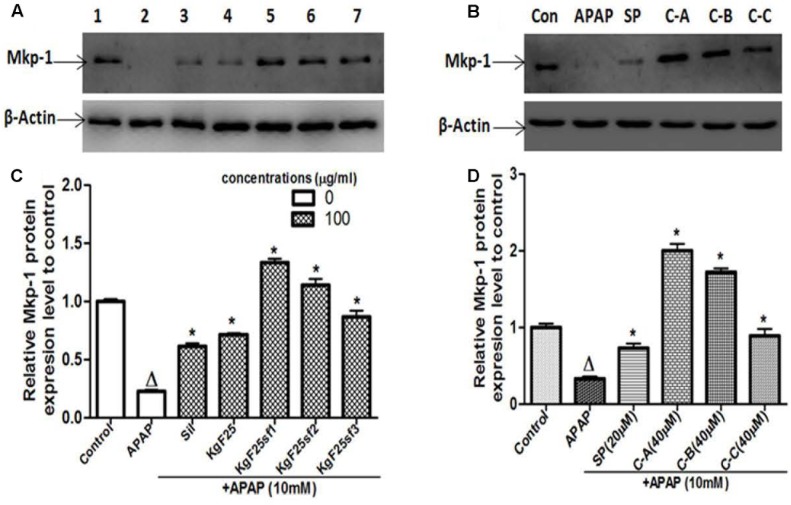
Active sub-fractions and isolated Limonoids up-regulated Mkp-1 protein expression. Cells were treated without or with APAP (10 mM), or co-treated with APAP (10 mM) and active sub-fractions or silymarin (100 μg/mL), isolated compounds (40 μM) or JNK inhibitor (20 μM) for 6 h. After treatment, total proteins were extracted from cells and Mkp-1 expression was determined by western blotting. β-actin was used as loading control. Each blot represents one of three independent experiments. **(A,B)** Effect of active sub-fractions and isolated compounds on Mkp-1 expression, respectively. **(C,D)** Densitometry analysis of blots, respectively, for active sub-fractions and isolated compounds. Values are means ± SD of three independent experiments in triplicate. ANOVA analysis: *F*(6,14) = 307.1, *P* < 0.0001 **(C)** and *F*(5,12) = 270.3, *P* < 0.0001 **(D)**. ^Δ^Values significantly different compared to control group (*P* < 0.05); ^∗^values significantly different compared to APAP intoxicated group (*P* < 0.05) using Bonferroni’s test. Lane1: control; Lane2: APAP; Lane3: silymarin+APAP; Lane4: KgF25+APAP; Lane5: KgF25sf1+APAP; Lane6: KgF25sf2+APAP; Lane7: Kgf25sf3+APAP. Sil: silymarin; KgF25: methylene chloride/methanol (75:25, v/v) fraction of *K. grandifoliola*; KgF25sf1: sub-fraction 1 of KgF25; KgF25sf2: sub-fraction 2 of KgF25; KgF25sf3: sub-fraction 3 of KgF25; SP: JNK inhibitor SP600125; C-A: 17-epi-methyl-6-hydroxyangolensate; C-B: 7-deacetoxy-7-oxogedunin; C-C: 7-deacetoxy-7R-hydroxygedunin.

Mkp-1, a phosphatase which preferentially inactivates the stress induced by JNK activation in most tissues, has been found to protect against APAP-hepatotoxicity (Hammer et al., 2006; Wancket et al., 2012). However, overexpression of ROS decreases its expression (Kamata et al., 2005). In search of the protective mechanism of isolated limonoids from *K. grandifoliola*, the Mkp-1 protein level has been evaluated by immunoblotting. As shown in **Figures 11A,B**, exposure of L-02 hepatocytes for 6 h resulted in a marked decrease of Mkp-1 protein level. Co-treatment with silymarin (100 μg/ml) (**Figure 11A**) or JNK inhibitor SP600125 (20 μM) (**Figure 11B**) only attenuated this decreasing. However, co-treatment with active sub-fractions (100 μg/ml) (**Figures 11A,C**) or isolated compounds (40 μM) (**Figures 11B,D**) markedly increased the expression of Mkp-1 by up-to 1.8-fold as compared to the untreated cells.

#### Limonoids of *K. grandifoliola* Induced Nuclear Translocation of Nrf2

**FIGURE 12:**
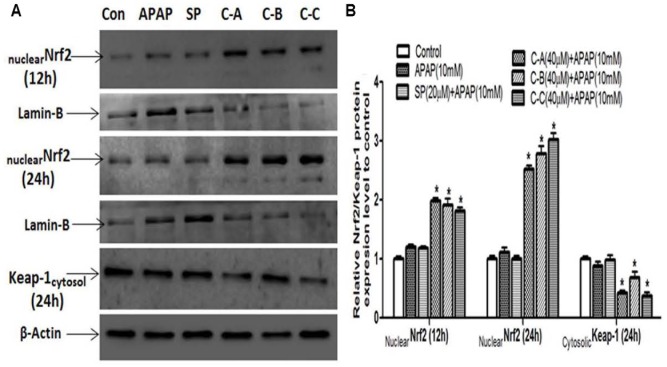
Isolated limonoids activated Nrf2-Keap-1 antioxidant defense system. Cells were treated without or with APAP (10 mM), or co-treated with APAP (10 mM) and isolated limonoids (40 μM) or JNK inhibitor (20 μM) for 12 and 24 h. After treatment, Nrf2 level was detected into the nuclear fraction and Keap-1 level was detected at 24 h into the cytosolic fractions by western blotting **(A)**. Lamin-B and β-actin were used as loading control, respectively, for the nuclear and cytosolic fraction. Each blot represents one of three independent experiments. **(B)** Densitometry analysis of blots. Values are means ± SD of three independent experiments in triplicate. ANOVA analysis: *F*(5,12) = 370.8, *P* < 0.0001 and *F*(5,12) = 48.80, *P* < 0.0001 **(B)**. ^Δ^Values significantly different compared to control group (*P* < 0.05); ^∗^values significantly different compared to APAP intoxicated group (*P* < 0.05) using Bonferroni’s test. SP: JNK inhibitor SP600125; C-A: 17-epi-methyl-6-hydroxyangolensate; C-B: 7-deacetoxy-7-oxogedunin; C-C: 7-deacetoxy-7R-hydroxygedunin.

Nrf2 plays a keys role in the cellular antioxidant defense system by regulating the transcriptional activation of various antioxidant enzymes (Jaiswal, 2004). Therefore, western blot analysis was performed to examine the ability of isolated limonoids to induce the nuclear translocation of Nrf2. As shown in **Figure 12A**, co-treatment of L-02 cells with limonoids from *K. grandifoliola* induced the translocation of Nrf2 into the nucleus by up to two and threefold, respectively, at 12 h and 24 h after treatment, as compared to untreated cells. There was no obvious change in nuclear Nrf2 level of APAP or JNK inhibitor treated cells. Interestingly, 24 h after treatment, the nuclear translocation of Nrf2 was correlated to a significant (*p* < 0.05) decrease of the expression of Keap-1 (an inhibitor of Nrf2 activation) into the cytosol in limonoids co-treated cells (**Figures 12A,B**).

### Limonoids from *K. grandifoliola* Increased mRNA Expression Levels of Antioxidants Enzymes

**FIGURE 13:**
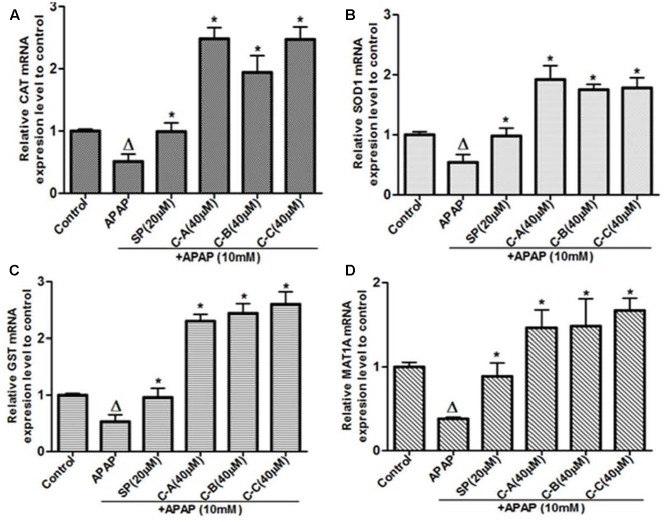
Limonoids from *K. grandifoliola* increased mRNA levels of CAT, SOD1, GST, and MAT1A genes. Cells were treated without or with APAP (10 mM), or co-treated with APAP (10 mM) and isolated limonoids (40 μM) or JNK inhibitor (20 μM) for 36 h. After treatment, RNA was extracted from cells and relative mRNA expression level of CAT **(A)**, SOD **(B)**, GST **(C)**, and MAT1A **(D)** were determined by qRT-PCR. GAPDH was used as internal control. Values are means ± SD of three independent experiments in triplicate. ANOVA analysis: *F*(5,12) = 70.43, *P* < 0.0001 **(A)**; *F*(5,12) = 45.72, *P* < 0.0001 **(B)**; *F*(5,12) = 113.0, *P* < 0.0001 **(C)**; and *F*(5,12) = 20.65, *P* < 0.0001 **(D)**. ^Δ^Values significantly different compared to control group (*P* < 0.05); ^∗^values significantly different compared to APAP intoxicated group (*P* < 0.05) using Bonferroni’s test. SP: JNK inhibitor SP600125; C-A: 17-epi-methyl-6-hydroxyangolensate; C-B: 7-deacetoxy-7-oxogedunin; C-C: 7-deacetoxy-7R-hydroxygedunin.

Gene’s expression studies are useful supplements to protein examination, as the mRNA levels represent a snapshot of the cell activity at a given time. Thus, the effect of isolated limonoids was evaluated by qRT-PCR on the expression of the antioxidant enzymes, CAT, SOD1, and GST chosen as target genes of Nrf2. In parallel, the expression of MAT1A was also evaluated. As presented in **Figure 13**, exposure of cells to 10 mM APAP for 36 h significantly reduced the mRNA levels of CAT, SOD1, GST, and MAT1A. In limonoids co-treated cells, the mRNA levels of CAT (**Figure 13A**) and GST (**Figure 13C**) were increased by up-to 2.8-fold as compared to the untreated cells. Similarly, the mRNA levels of SOD (**Figure 13B**) and MAT1A (**Figure 13D**) were also increased by up-to 1.77-fold.

## Discussion

Previous studies conducted have shown the hepatoprotective potential of *K. grandifoliola* and a highly active fraction has been isolated (Njayou et al., 2015, 2016). The main purpose of the present study was to identify compounds from the plant bearing hepatoprotective activity and the related molecular mechanism against APAP-induced hepatotoxicity in normal human liver L-02 cells. The results showed the protective action of three isolated known limonoids (17-epi-methyl-6-hydroxylangolensate, 7-deacetoxy-7-oxogedunin and deacetoxy-7R-hydroxygedunin) which was evidenced not only by their ability to alter the critical events in APAP-cell death mechanism, but also by their capacity to improve the cellular antioxidant defense system.

Acetaminophen continues to serve as an important model of DILI for phytotherapeutics and other natural compounds (Jaeschke et al., 2011, 2013). The hepatotoxicity of APAP is initiated firstly by its bio-activation to form a reactive metabolite NAPQI, and overexpression of CYP2E1 plays a critical role in this process (Jaeschke et al., 2002). Accumulated NAPQI depletes GSH pool due to the saturation of both glucuronidation and sulfatation pathways (Hinson et al., 2010); leading to excessive generation of ROS which subsequently triggers the process of lipid membrane peroxidation and causes destruction of cells components and cell death (Jaeschke et al., 2002; Schnackenberg et al., 2009). In this study, APAP was used at the determined concentration of 10 mM. This concentration was found to be toxic for L-02 hepatocytes after 36 h of incubation leading to about 50% decrease of cell viability and great increase of ALT leakage into the incubation medium (**Figure 2**). These observations could be the consequence of metabolic activation of APAP as proven by the overexpression of CYP2E1 (**Figures 6A,B**), depletion of cellular GSH (**Figure 6E**), overproduction of ROS (**Figure 7A**) and increase formation of MDA (**Figure 7B**) when L-02 hepatocytes were incubated in presence of APAP alone. However, co-treatment of cells with active sub-fractions, isolated limonoids or JNK inhibitor dose-dependently prevented cell death and ALT leakage into the incubation medium (**Figure 4**). Likewise, significant inhibition of CYP2E1 expression (**Figures 6C,D**), restoration of cellular GSH (**Figure 6E**), attenuation of ROS generation (**Figure 7A**) and inhibition of lipid peroxidation (**Figure 7B**) were observed in limonoids co-treated cells. These results suggest that limonoids from *K. grandifoliola* protect L-02 hepatocytes against APAP-induced oxidative injury. Similarly, lupeol, a triterpenoid compound found in many plant species including mango, olive and fig was evidenced to protect primary rat hepatocytes against APAP-induced oxidative damage through inhibition of GSH depletion, ROS production and lipid membrane peroxidation (Kumari and Kakkar, 2012b).

The second hit which plays a major role during APAP cell death mechanism has been well established as JNK activation (Gunawan et al., 2006; Hanawa et al., 2008; Saito et al., 2010). In fact, early oxidant stress which occurs after GSH depletion initiates the activation of various MAP Kinases. The activities of different kinases converge to the phosphorylation of JNK, which then translocates into the mitochondria and amplifies the mitochondrial oxidant stress (Nakagawa et al., 2008; Saito et al., 2010). This mitochondrial oxidant stress leads to the opening of the membrane permeability transition (MPT) pore with collapse of the membrane potential and cessation of ATP synthesis. In addition, opening of MPT pore causes mitochondrial Bax translocation and mitochondrial matrix swelling, with the rupture of outer membrane and the release of intermembrane proteins, such as endonuclease G and AIF, which translocate into the nucleus and induce DNA damage (Bajt et al., 2006, 2008). Taking together, these events have been recognized as the main cause of cell death (Bajt et al., 2011; Jaeschke et al., 2012; Xie et al., 2014). In support of these events in our study, phosphorylation of JNK (**Figure 8**), mitochondrial translocation of p-JNK (**Figure 9**) and Bax (**Figure 10**), and release of AIF into the cytosol (**Figure 10**) were observed in APAP-treated L-02 hepatocytes. All these parameters were effectively attenuated when cells were co-treated with isolated limonoids and their effects were comparable to those observed in JNK inhibitor co-treated cells. These findings suggest that inhibition of JNK activation contributes to the protective effect of these limonoids. These results are also in agreement with previous reports documenting that pharmacological inhibition of JNK phosphorylation or the silencing of JNK gene expression resulted in reduced liver injury after APAP overdose (Gunawan et al., 2006; Henderson et al., 2007; Hanawa et al., 2008).

Since previous studies have demonstrated that JNK activation is a key event that perpetuates hepatocellular damage during APAP hepatotoxicity (Gunawan et al., 2006; Hanawa et al., 2008; Saito et al., 2010), we assessed the effect of isolated limonoids on the expression of Mkp-1, a primary phosphatase responsible for dephosphorylating JNK (Keyse, 2000; Hammer et al., 2006). It has been reported that Mkp-1 protects mice against APAP-hepatotoxicity (Wancket et al., 2012). However, excess ROS generated during toxic hepatic injuries inhibits Mkp-1 and prolongs JNK activation which perpetuates hepatic damage (Kamata et al., 2005; Boutros et al., 2008; Wancket et al., 2012). In this study, we observed a significant decreased of Mkp-1 protein level (**Figures 11A,B**) in APAP treated L-02 hepatocytes which effectively correlates with overproduction of ROS and sustained JNK activation. Co-treatment of cells with JNK inhibitor SP600125 only attenuated this decreasing (**Figure 11B**). In contrast, when cells were co-treated with isolated limonoids, Mkp-1 protein levels were increased by up-to 1.8-fold as compared to untreated cells. These findings suggest that inhibition of JNK activation may be due to the up-regulation of Mkp-1 expression which could therefore contributes to protect L-02 hepatocytes against APAP-toxicity.

The transcription factor Nrf2, which regulates the transcriptional activation of various antioxidant enzymes has been reported to play a key role in protecting against hepatotoxicity of several chemicals (Gum and Cho, 2013; Pang et al., 2016). Under normal condition, Nrf2 is located into the cytosol, where it forms an inactive complex with it repressor Keap-1 (Jaiswal, 2004). Upon stimulation, Nrf2 dissociates from Keap-1, translocates into the nucleus where it binds to ARE and promote the expression of antioxidant enzymes (Niture et al., 2010). Therefore, up-regulation of Nrf2 in the nucleus can result in a reduction of ROS, and correspondingly, protects cells against APAP-toxicity. In our study, immunoblotting analysis in cells treated with APAP alone or co-treated with JNK inhibitor showed no obvious changes neither in nuclear Nrf2 protein level after 12 h or 24 h of treatment nor in cytosolic Keap-1 protein level after 24 h of treatment (**Figure 12**). However, in limonoids co-treated cells, the nuclear protein level was increased by up to two and threefold, respectively, at 12 and 24 h after treatment. This effect was correlated with a significant (*p* < 0.05) decrease of cytosolic keap-1 protein level (**Figure 12**). Nrf2 target genes include antioxidant and detoxification enzymes as CAT, SOD1, and GST (Jaiswal, 2004). GST uses GSH to detoxicate the xenobiotic or their reactive metabolites in the liver. SOD dismutase superoxide anions into hydrogen peroxide (H_2_O_2_). Then, CAT metabolizes H_2_O_2_ to O_2_ and H_2_O (Kaiiska et al., 2000; Dai et al., 2006). The expression of these enzymes decreases during APAP overdose (Kumari and Kakkar, 2012a). In the present study, CAT, SOD and GST mRNA levels were significantly (*p* < 0.05) reduced in L-02 cells in response to APAP treatment alone (**Figure 13**). In contrast, CAT, SOD, and GST mRNA levels were significantly (*p* < 0.05) increased in limonoids co-treated cells. Taking together, these observations suggest that limonoids from *K. grandifoliola* stimulate Nrf2, which then dissociates from Keap-1, translocates into the nucleus and enhances the expression of CAT, SOD, and GST, and contributes therefore to the protection of L-02 hepatocytes against APAP-toxicity.

Methionine adenosyltransferase-1A is the enzyme responsible for the synthesis of *S*-adenosyl-L-methionine (SAM), the main precursor of GSH in the liver (Lieber, 2002). Previous studies report that depletion of the liver GSH content induced by various hepatotoxins is largely due to the decrease of MAT1A expression (Brown et al., 2010; Cederbaum, 2010). In our study, the significant (*p* < 0.05) decrease of MAT1A mRNA level (**Figure 13D**) observed in APAP-treated cells effectively correlated with the depletion of cellular GSH. Co-treatment of cells with limonoids significantly (*p* < 0.05) increased MAT1A mRNA level. These data suggest that restoration of GSH observed in limonoids co-treated cells could be due to the increase expression of MAT1A.

On a structural basis, limonoids isolated in this study are related to two groups. Compound A (17-epi-methyl-6-hydroxylangolensate) is a derivative of methylangolensate while compound B (7-deacetoxy-7-oxogedunin) and C (deacetoxy-7R-hydroxygedunin) derive from gedunin. More than one hundred different natural limonoids have been isolated and are of diverse structural and biological activities that encompass antiparasitic, anticancer, antioxidant, anti-inflammatory, neuroprotective, antiplasmodial, antibacterial… (Tundis et al., 2014). Methylangolensate has been demonstrated to inhibit NF-kB signaling pathway while a degradation product of limonoids, Fraxinellone exhibits anti-inflammatory activity via inhibition of Ik-B kinase (IKK) and extracellular signal-related kinase (ERK1/2) phosphorylation without affecting c-Jun N-terminal kinase (JNK1/2) and p38 phosphorylation (Kim et al., 2009). Compounds B and C which are structurally close possess antiproliferative activity through modulation of the 90-kDa heat shock protein (Tundis et al., 2014). Due to the great diversity of the structure and biological activities of limonoids, extensive studies are needed in order to relate the structure type to the molecular activities. However, this study provides for the first time evidence of the action of two structural diverse limonoids on the signaling pathway of APAP-induced hepatotoxicity in L-02 cells line through up-regulation of Mkp-1, an endogenous inhibitor of JNK phosphorylation, and nuclear translocation of Nrf2, a transcription factor that regulates the expression of numerous antioxidant enzymes.

## Conclusion

Our findings demonstrated that limonoids isolated from *K. grandifoliola* protect normal human liver L-02 cells against APAP-induced hepatotoxicity mainly through induction of Mkp-1 and nuclear translocation of Nrf2. Also, further analysis including *in vivo* and toxicological studies are needed to select the most potent compound that may be useful as therapeutic agents against DILI.

## Author Contributions

AK, FN, and PM defined the research subject and its aims, conceived and designed the experiments. GG, FY, and PM provided facilities to perform the work. AK, RT, HH, FS, and BO prepared the compounds and performed the experiments. AK, FN, FY, and GG analyzed the data and wrote the paper. All the authors read and approved the final version of this manuscript.

## Conflict of Interest Statement

The authors declare that the research was conducted in the absence of any commercial or financial relationships that could be construed as a potential conflict of interest.

## Abbreviations

AIF: apoptosis-inducing factor
ALT: alanine aminotransferase
APAP: acetaminophen
Bax: Bcl_2_-associated X protein
CAT: Catalase
CYP2E1: cytochrome P450 2E1
DILI: drug-induced liver injury
GSH: reduced glutathione
GST: glutathione-*S*-transferase
H_2_DCFDA: 2′-7′-Dichloro-dihydrofluorescein diacetate
HPLC: high performance liquid chromatography
HRMS: high resolution mass spectrometry
JNK: c-Jun N-terminal Kinase
Keap-1: Kelch-like ECH-associated protein-1
KgF25: methylene chloride/methanol (75:25 v/v) fraction of *K. grandifoliola*
KgF25sf: sub-fraction of KgF25
MAPK: mitogen-activated protein kinase
MAT1A: methionine adenosyltransferase-1A
Mkp-1: mitogen-activated protein kinase phosphatase-1
NAPQI: *N*-acetyl-para benzo-quinone imine
NMR: nuclear magnetic resonance
Nrf2: nuclear factor erythroid 2-related factor 2, p-JNK: Phospho-JNK
ROS: reactive oxygen species
SOD1: superoxide dismutase-1
SP: JNK inhibitor SP600125
