# Supplementary material for: Induction of Mkp-1 and Nuclear Translocation of Nrf2 by Limonoids from Khaya grandifoliola C.DC Protect L-02 Hepatocytes against Acetaminophen-Induced Hepatotoxicity
Source: Front Pharmacol. 2017 Sep 19;8:653. doi: 10.3389/fphar.2017.00653 (PMC5610691; doi:10.3389/fphar.2017.00653)
Supplement: Supplementary file 1 [file Data_Sheet_1.DOC]

**Supplementary file**


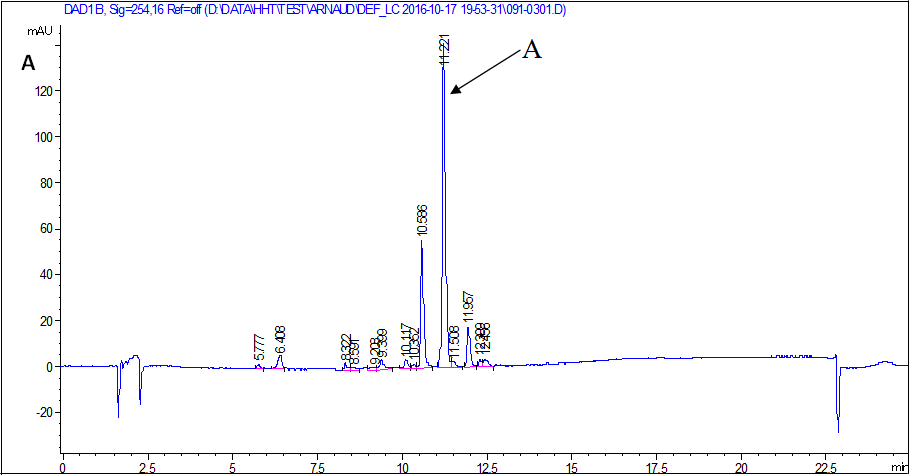


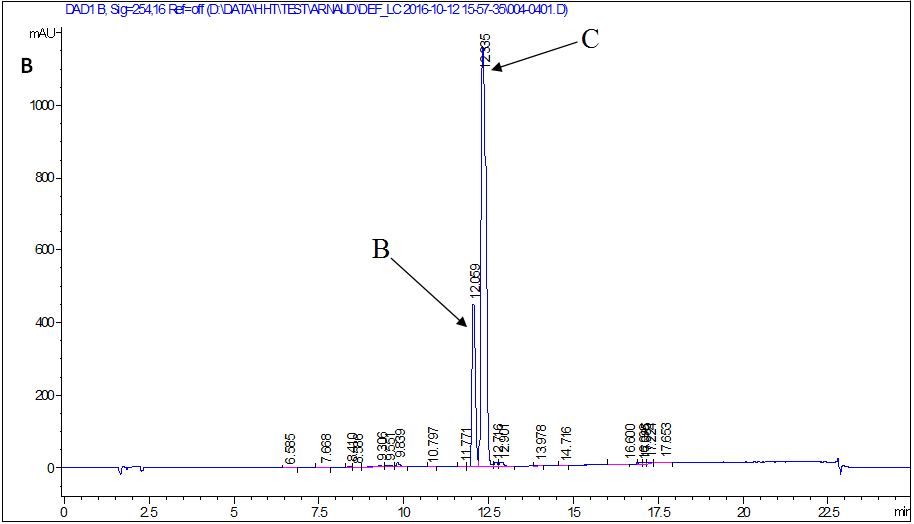
**Figure S1: HPLC chromatogram of selected active sub-fractions;** **A** and **B**, chromatogram of KgF25sf1 and KgF25sf2 respectively analyzed by HPLC-ACN-Standard-Method. Eclipse XDB-C8 column (9.4 × 250 mm, 5 µm particle size); mobile phase: (A) water and (B) acetonitrile; elution condition: B, 0-15 min, increasing gradient from 0 to 30 % B, 15-20 min, linear gradient 100 % B; 20-25 min, linear gradient 30 % B; flow rate: 1 mL/min; injection volume: 5 µL. KgF25: methylene chloride/methanol (75:25, v/v) of *K. grandifoliola*; KgF25sf1: sub-fraction 1 of KgF25; KgF25sf2: sub-fraction 2 of KgF25.

**Figure S2:** ^1^H NMR spectrum of compound **A** measured at 600 MH_Z_ in DMSO

**Figure S3:** ^13^C NMR spectrum of compound **A** measured at 150 MH_Z_ in DMSO

**Figure S4:** ^1^H NMR spectrum of compound **B** measured at 600 MH_Z_ in DMSO

**Figure S5:** ^13^C NMR spectrum of compound **B** measured at 150 MH_Z_ in DMSO

**Figure S6:** ^1^H NMR spectrum of compound **C** measured at 600 MH_Z_ in DMSO

**Figure S7:** ^13^C NMR spectrum of compound **C** measured at 150 MH_Z_ in DMSO

**Figure S8:** HRMS spectrum of compound **A**

**Figure S9:** HRMS spectrum of compound **B**

**Figure S10:** HRMS spectrum of compound **C**
